# A road map for point-of-care ultrasound training in internal medicine residency

**DOI:** 10.1186/s13089-019-0124-9

**Published:** 2019-05-09

**Authors:** Charles M. LoPresti, Daniel J. Schnobrich, Renee K. Dversdal, Frank Schembri

**Affiliations:** 1 Division of Medicine, Louis Stokes Cleveland Veterans Affairs Medical Center, 10701 East Boulevard, Cleveland, OH 44106 USA; 2 Case Western Reserve University School of Medicine, 2109 Adelbert Rd, Cleveland, OH 44106 USA; 3 Department of Medicine, University of Minnesota, 401 East River Parkway, Variety Club Research Center, Suite 131, Minneapolis, MN 55455 USA; 4 Department of Medicine, Oregon Health & Science University, 3181 SW Sam Jackson Park Road, Portland, OR 97239-3098 USA; 5 Department of Medicine, Boston Medical Center, 715 Albany St. E-113, Boston, MA 02118 USA

**Keywords:** Point-of-care, Education, Internal medicine, Residents

## Abstract

**Background:**

Ever-expanding uses have been developed for ultrasound, including its focused use at the bedside, often referred to as point-of-care ultrasound (POCUS). POCUS has been well developed and integrated into training in numerous fields, but remains relatively undefined in internal medicine training. This training has been shown to be desirable to both educators and trainees, but has proven difficult to implement. We sought to create a road map for internal medicine residency programs looking to create a POCUS program.

**Results:**

Four internal medicine residency programs that have successfully integrated POCUS training describe their programs, as well as the principles and concepts underlying program development and execution. Review of educational teaching and assessment methods is outlined, as well as suggestions for integration into an already busy residency curriculum. Commonly reported barriers to POCUS implementation such as faculty development, equipment purchasing, resident supervision and quality assurance are addressed. Specific POCUS applications to target are touched upon, and a comparison of applications taught within these four programs suggest that there may be enough similarities to suggest a common curriculum. Finally, future needs are discussed.

**Conclusions:**

POCUS can be successfully taught to internal medicine residents as a part of internal medicine training. Many common elements and principles are evident on review of these four described successful programs. Future support, in the form of endorsed medical society guidelines, will be needed before POCUS is universally incorporated across internal medicine residency training programs.

## Background

Since it first came into use in the 1960s, medical ultrasound has undergone innumerable improvements and evolutions that have created a tremendously versatile and powerful technology. In addition to its uses by sonographers to create comprehensive images for radiologists, obstetricians, and cardiologists, it is also heavily utilized to increase the safety of procedures, and over the past several decades, point-of-care ultrasound (POCUS) has become increasingly popular. POCUS refers to a focused, often dichotomous use by the treating provider at the bedside, which can be immediately integrated into management decisions. First popularized in emergency medicine, more recently there has been uptake of POCUS in both general and subspecialty internal medicine.

Despite these trends, structured POCUS curricula have only recently started coming into existence in internal medicine residency training [1]. Many IM program directors feel that formal POCUS education should be included in IM residency training, and many learners desire training, but numerous barriers exist including both a lack of formal guidelines and experience by most programs in teaching these skills [2]. In this document, we will outline what is needed to set up and execute a POCUS program to teach a foundational set of skills to internal medicine residents based upon the experience of four successful programs.

## Results

### Training in point-of-care ultrasound

A comprehensive training program in POCUS should ideally be competency based and parallel training of other bedside evaluations and diagnostics. Such a program may involve a pre-course need and knowledge assessment, a variety of teaching methods, a post-course evaluation and mechanisms to assess continued competency and quality assurance.

#### Timing and length of training

Adding POCUS education to an already dense IM curriculum can be a challenging task, and its timing and length may be influenced by the extent of the curriculum to be delivered as well as practical considerations such as the number of available trained faculty, amount of time learners are accessible, and access to resources such as simulation for task training and scanning practice. While some programs have described their brief experiences teaching one discrete POCUS application, numerous IM programs teaching more comprehensive skills have dedicated roughly a 1- to 2-week period to introductory training followed by intermittent reinforcing sessions [3, 4]. In one report, Schnobrich et al. described a 30-h introductory course delivered over 5 days during intern orientation in which learners showed a statistically improved subjective and objective assessment of their ultrasound knowledge and skills in a wide range of skills [5]. Similarly, programs affiliated with Case Western Reserve, Boston University and Oregon Health & Science University have briefer introductory components of ultrasound use during or after intern orientation, but offer subsequent PGY2/3 electives. In all four programs, resident response to these formal curricula have been very positive and demands for more further formal instruction have been universally very strong.

A more frequent longitudinal component to didactic instruction may be beneficial, if sometimes more challenging to implement. Kelm et al. described IM-based training with a shorter initial training period (~ 4 h), but with a subset of residents participating in a longitudinal component involving monthly ultrasound-based morning reports and afternoon ultrasound rounds [6]. The authors found that the group participating in the ongoing training was more likely to correctly identify ascites, renal pathology and pleural effusion on static ultrasound images, and concluded that the addition of a longitudinal component to ultrasound education may result in improved knowledge retention. Regardless of how introductory and basic didactics are delivered, subsequent opportunities to scan patients on the ward or in clinic, integrate findings into management, and receive feedback are highly important to all programs.

#### Educational methods

A comprehensive POCUS program can be most effective when involving a variety of teaching methods. Many programs utilize some combination of online/in person didactic lectures, pre- or post-course quizzes, live models, simulation task trainers, dedicated ultrasound trainers, and direct patient scanning (Table 1). In several studies, residents were asked to rate the helpfulness of each of the above teaching modalities. IM residents found scanning live models and/or hospitalized or clinic patients, along with didactic lectures to be most helpful. Interestingly, taking and reviewing quizzes were rated as a less effective educational strategy [1, 5].

**Table 1:** Educational methods in POCUS training

Educational method	Utility
Didactic lectures	Effective for new learners to process the basics of ultrasound, knobology, introductions to specific applications. An interactive approach is helpful, with immediate demonstration of concepts on an ultrasound machine. Online didactics may be used to save instructor time
Procedural task trainers	Individual procedure-oriented trainers such as central line, thoracentesis and paracentesis mannequins, as well as ultrasound-compatible gel-blocks for IV placement
Ultrasound trainers	More costly, comprehensive ultrasound models manufactured by several companies capable of replicating the scanning experience, image acquisition and interpretation. Ultrasound trainers can be used to display idealized normal anatomy, or a variety of expandable pathologies
Live models	Represent a good resource, especially for students immediately after initial didactics. These can be standardized patients or individual learners who are part of the course (if comfort level permits) and are usually used to achieve standard views with normal anatomy. Often rated highly by students
Direct patient scanning	A powerful way for learners to solidify their knowledge. Usually positioned after learners acquire basic image acquisition and interpretation skills, it can be structured as a known or unknown assessment to increase challenge. Often very highly rated by students
Individual portfolio creation	A method to allow continued, independent learning. The learner acquires a collection of saved exams, which is later reviewed and appraised by an instructor

#### Resident supervision/continued quality assurance

Many medical specialties perform POCUS tailored to their practices, and each is responsible for defining adequate training, competency, and quality assurance in their fields. The American Medical Association (AMA) policy H-230.960 specifically states that the use of ultrasound is not the intellectual property of any one specialty, and each specialty must define for themselves their scope of practice. With this guidance, a comprehensive ultrasound program for IM residencies should include defined, IM-specific competencies with subsequent regular assessment. Like other ACGME patient care-related milestones, this should involve direct trainee assessment, often including the creation of a portfolio for image review and approval. In its current state, many programs will “sign off” on resident ability to use POCUS for procedural guidance, however, diagnostic POCUS is more nuanced, and rigorous quality assurance systems need to be in place prior to granting residents the ability to make clinical decisions on their own.

It is the responsibility of all teaching programs to provide adequate supervision to medical trainees for all of their professional activities. This supervision may be direct or indirect, and is ideally tailored to the learners’ demonstrated proficiency, the activity being performed, and institutional resources. This supervision may often be quite challenging for internal medicine training programs, as the majority of teaching faculty are not likely to be proficient themselves. We are aware of a variety of mechanisms employed by teaching programs including partial delegation of supervisory roles to chief residents or other trained educators, the use of image review middleware to provide learners rapid feedback away from patient care, and formalization of entrustment using tools such as the CLUE-CEX exam or a formal entrustable professional activity [7].

### Point-of-care ultrasound applications

Several factors should be considered when choosing which POCUS applications to teach internal medicine residents. These include the amount of curricular time available, perceived usefulness of the applications, ability of the program to provide opportunities to learn these skills, and evidence that POCUS users can attain these skills in the time available. While no standard curriculum yet exists to guide which application or guidelines should be taught to IM residents, we note a striking agreement amongst the programs at each of our institutions despite being developed independently (Table 2).

**Table 2:** Core skills taught with emphasis in each residency program

	CWRU	UMN	OHSU	BU
*Procedural guidance*				
Central venous catheterization	+	+	+	+
Paracentesis	+	+	+	+
Thoracentesis	+	+	+	+
Arthrocentesis	+	−	−	−
Lumbar puncture	#	−	#	−
Peripheral access	+	−	−	+
Peripherally inserted central catheter (PICC)	−	−	−	−
*Cardiac*				
Pericardial effusion	+	+	+	+
Left ventricular contractility	+	+	+	+
Right ventricular enlargement	+	+	+	+
Chamber size/wall thickness	#	+	#	+
Severe valvular abnormalities	#	−	−	+
Other valvular abnormalities	−	−	−	−
Right atrial pressure (IVC)	+	+	+	+
Wall motion abnormalities	#	−	−	#
*Pulmonary*				
Pleural effusions	+	+	+	+
Pulmonary edema	+	+	+	+
Consolidation	+	+	#	+
Pneumothorax	+	+	+	+
*Abdomen*				
Ascites	+	+	+	+
Bladder volume	+	+	+	+
Hydronephrosis	+	+	+	+
Organomegaly	#	+	#	−
AAA	+	−	#	#
Gallbladder	#	−	−	#
*Vascular*				
Lower extremity DVT	+	−	#	+
*Musculoskeletal*				
Cellulitis/abscess	+	+	+	+
Muscle/tendon tears	−	−	−	−
Joint effusions	+	−	−	−
Fracture	#	−	−	−
*Ophthalmologic*				
Optic nerve sheath diameter	−	−	−	−

Additional skills may be presented as time and interest allows

*CWRU* Case Western Reserve University, *UMN* University of Minnesota, *OHSU* Oregon Health & Science University, *BU* Boston University

“+”: Skill taught. “-”: Skill not addressed. “#”: Skill is demonstrated, but not with the intent for resident use

Literature and precedent from emergency medicine (EM) are useful in determining the didactic time needed to teach various applications. Simple applications, such as evaluation for abscess, may be taught in as little as 30 min, while more complicated ones, such as cardiac or gallbladder, may take 4–6 h, plus significant additional time for mentored scanning [8, 9]. Notably, EM guidelines specify 16–25 h of didactic time followed by mentored and supervised scanning to teach 6–10 core applications, which has become an accepted standard for demonstrating experience and for credentialing [10]. EM guidelines also note that skills sets may transfer from one application to another, and thus there may be some benefit to teaching multiple skills [9].

Despite the many career paths of IM residents, most programs agree that a core of predominantly cardiac, pulmonary, and abdominal applications should be taught, and may additionally serve as a platform to build advanced skills upon. This is supported by surveys of internal medicine program directors [11], practicing internists [12], and internal medicine residents [2] which suggest that these skills are generally considered the most useful.

A final major consideration is to choose applications that the literature supports can be accomplished by most or all learners. For example, it is generally agreed that POCUS users with core training and 25–50 mentored scans can sufficiently evaluate for pericardial effusions, left ventricular contractility, and right heart function [10]. Other findings, such as most non-severe valvular abnormalities and wall motion abnormalities appear to be detected much less reliably at this level of training. While there may be value in demonstrating more advanced skills, it should be clear to all learners which skills can be reliably acquired during the training the residency can offer to prevent inappropriate use. The optimal ultrasound skill set is likely to further evolve as internists further develop its use in internal medicine practice, and should be continuously re-evaluated.

### Resources and interdepartmental collaboration

To build a successful POCUS curriculum, IM residency programs require several categories of resources including equipment, faculty expertise, didactic content, hands-on teaching personnel, and a means of formative and summative assessment.

#### Equipment

As technology has evolved, POCUS equipment options have increased. Lower priced units have become available, including hand-held units to purchase or lease. These hand-held units are popular for their ease of transportation and use, however, present challenges for early learners given reduced screen size. Many suggest machines designed specifically for POCUS clinicians, as they often have a more approachable interface while losing little important functionality compared to machines designed for sonographers. Many manufacturers offer discounted educational pricing for educational-designated machines.

At a minimum, each program needs one dedicated machine configured with a linear, phased array and, if cost permits, a curvilinear probe. Maximizing access to POCUS machines encourages more frequent use, for example providing a cart-based unit for each floor and a hand-held for each resident team.

Some programs have access to simulation centers or ultrasound labs with machines that can be used by all learners. These spaces, when available, provide an arena for alternating didactics and hands-on sessions with normal models for the early learning phases. As learners progress, access to machines in clinical spaces is paramount for ongoing image acquisition and interpretation skill development, as actual patients provide both technical practice and the benefit of developing clinical integration skills.

Finances are often a limitation, and various methods have been used to acquire machines, including using medical school or hospital simulation centers (CWRU, UMN, OHSU, BU), noting the necessity of equipment if residents are expected to perform procedures (CWRU, UMN, OHSU, BU), quality improvement grant awards (UMN), use of machines destined for surplus from other divisions, group purchasing discounts when large departments/divisions are buying new machines, and making a return on investment (ROI) business case for ultrasound training later producing increases in billing or savings from enhanced patient safety (CWRU, BU).

Once machines are accounted for, there are other equipment considerations. If the assessment or quality assurance plan includes image portfolio collection and review, there should be a process to offload images in a HIPAA compliant fashion. Many machines come capable of connecting via wireless internet and archiving directly to image archival systems or the electronic medical record, however, others are not and require a “middleware” solution to sync images/clips to a secure on-site or cloud-based server. Some sites with student and resident learners decide not to have every educational image uploaded to the clinical spaces, and instead use “middleware” to differentiate between clinical and educational images. To save images, on-site requires the existence of a secure and sizeable server, which may already exist in other departments. Alternatively, a cloud-based server option can be added along with the “middleware” as a monthly operational cost.

#### Faculty

The availability of ultrasound-skilled faculty to lecture provides hands-on education and creates ongoing curricula which can be a challenge. It is imperative to have a faculty “champion” who leads the development and maintenance of the program. Several successful programs have appointed a “Director of IM Ultrasound Education” or similar role with protected time, averaging 0.15–0.25 FTE.

Besides having a faculty champion, other faculty must become involved to continuously promote the program. Reaching out to other willing educators is critical as hands-on scanning sessions require at most 5 learners per teacher, and ideally 3–4. Interdepartmental and interprofessional collaboration have been invaluable at each of our institutions in this effort. Sonographers and echocardiographers have been excellent teachers for image acquisition skills. Several universities have found dedicated Educational Sonographers to be a core asset for their programs. In addition, we encourage growing programs to collaborate with other POCUS users within their institutions, in departments such as EM, Pulmonology/Critical Care, Cardiology, Family Medicine, Rheumatology and Anesthesiology, amongst others.

## Discussion

While some programs have quickly adapted POCUS training into traditional IM residency training, it remains relatively new, and there are many areas where growth and development are needed. Existing IM POCUS curricula are influenced heavily by well-documented EM training curricula. While this provides a good starting point, there are significant differences that make this extrapolation from EM to IM training less than ideal. IM residents see less unique patients per week than EM residents do, which inherently provides fewer opportunities to use POCUS, and may affect learning curves. Adding to the challenge, internal medicine residents typically learn from a much larger pool of faculty than many emergency medicine residents, making the direct training to proficiency of all teaching faculty difficult. Outcome data will be needed on the effectiveness of curricula to ensure that residents are learning, and effectively retaining this knowledge, and applying it correctly to real-world patient care. Given the already dense curriculum and time limitations of IM residency, innovative curriculum will need to be developed that works synergistically with what is already in place.

Further demonstration of techniques for supervision of resident POCUS use after initial training is needed, as residents are unlikely to be ready for completely independent practices after their initial training programs. Solutions to these issues are likely to need to be customized by each institution, and may overlap somewhat with those offered to attending physicians seeking to gain proficiency.

We feel that it is important to note that what we have outlined above amounts to opinion on what makes for a foundational POCUS program. The actual scope of practice for POCUS has not been defined, and outcomes research may help more accurately establish the appropriateness of various POCUS applications for the IM community.

Additionally, to make high-quality POCUS training more generalizable to all IM residency programs, it will be crucial that there is formal support and guidance from major medical societies governing internists. This was found to be a crucial catalyst for education, research, quality assurance and scholarship when undertaken by the American College of Emergency Physicians during emergency ultrasound’s POCUS infancy. Formal support with guidelines on POCUS programs will allow individual programs to overcome many of the barriers they currently face.

Finally, it is important to note that the POCUS movement is not limited to the GME community. POCUS training is flourishing in the undergraduate medical education community as well. These POCUS-trained medical school graduates contend with entering an IM GME world currently unprepared to support their continued POCUS growth and use. It is imperative that the internal medicine community continues to develop POCUS curriculum and move towards standardization of POCUS training to ensure safe and high-quality use in the years to come.

## Conclusions

POCUS can be successfully taught to internal medicine residents as a part of internal medicine training. Many common elements and principles are evident on review of these established programs (Table 3).

**Table 3:** Outline of POCUS program components at 4 institutions

	CWRU	UMN	OHSU	BU
Timeline of curriculum	PGY-1: 2 half day mixed didactic and hands-on sessions *Implemented 2013* PGY-2/3: optional 2-week elective (30 h/week) *Implemented 2015* ALL: interspersed hour long didactics throughout the 3 years of training (based on faculty availability) *Implemented 2015*	PGY-1: 25 h of training during intern orientation (all interns) *Implemented 2012* PGY-2/3: optional 40 h “advanced course” *Implemented 2014* Procedural service also available as a 2-week elective *Implemented 2016*	PGY-1: 3 half day mixed didactic and hands-on sessions *Implemented 2014* PGY-2/3: optional 2 to 3-week elective (30 h/week) *Implemented 2014*	PGY 1: ultrasound-guided procedural training during orientation (8 h) plus several sessions throughout the year (6 h) + OSCE *Implemented 2012* PGY-2/3: optional 1 week elective (36 h) *Implemented 2014* Procedural service which relies heavily on ultrasound also available as elective (40 h/week) *Implemented 2007*
Primary educational methods	Combination of didactics with supervised scanning using task trainers, ultrasound simulators and live ultrasound models For optional advanced elective, direct scanning of hospitalized patients	Intro course: largely web-based video didactics with quizzes, extensively uses of scanning of models, use of ultrasound simulators, and procedural task trainers Advanced course uses similar techniques, with addition of 25 h of scanning patients on wards	Didactic lectures alternating with hands-on scanning practice. Uses ultrasound simulators, and procedural task trainers	Didactic lectures followed by task trainers for procedures. Practice scanning on learners and ultrasound simulator followed by bedside patient scanning
Equipment	Multiple dedicated laptop and hand-held machines on wards and in simulation center available for resident use (hospital funded)	Multiple dedicated laptop machines on wards (hospital funded) and in simulation center (university funded)	Multiple dedicated laptop machines on wards (some hospital, some university funded) and simulation center (university funded). Have not yet received requested funding for hand-held units	Multiple laptop and larger machines based in ICU with loans to floor, and simulation center (all hospital funded)
Image management	Images uploaded to a dedicated network drive	Residents may keep portfolios and submit interpretations submitted manually starting with advanced course Process to use PACS and EMR in development	Middleware (see resources paragraph)	Locally stored on machines Residents may keep portfolios and submit manually as part of 1 week ultrasound elective
Quality assurance	Resident image review occurs from faculty supervision at the time of image acquisition	No formal system specific to residents. Exists in many clinical departments that residents rotate through	Weekly image review sessions for residents on elective, clinical image QA varies by department	No formal system. Confirmatory studies highly encouraged/stressed during didactics
Primary faculty involved	Hospitalists	Hospitalists, critical care	Hospitalists, emergency medicine, anesthesia	Critical care, cardiology, ER staff (case review during elective), internists at early stages

*CWRU* Case Western Reserve University, *UMN* University of Minnesota, *OHSU* Oregon Health & Science University, *BU* Boston University
